# Efficient Terpene Production by Marine Thraustochytrids: Shedding Light on the Thermodynamic Driving Force

**DOI:** 10.1128/mBio.01976-21

**Published:** 2021-09-28

**Authors:** Natalja Kulagina, Jennifer Perrin, Sébastien Besseau, Vincent Courdavault

**Affiliations:** a Université de Tours, EA2106 “Biomolécules et Biotechnologies Végétales,” Tours, France

**Keywords:** thermodynamics, metabolic flux, squalene, terpene engineering, *Aurantiochytrium* sp., thermodynamics

## Abstract

Terpenoids, such as squalene, are valuable compounds for cosmetic and drug industries, the supply of which is often limited by natural sources. Alternative production strategies have been investigated for decades but remain challenging due to low yields. In a recent study, Zhang and coworkers (A. Zhang, K. Mernitz, C. Wu, W. Xiong, et al., mBio 12:e0088121, 2021, https://doi.org/10.1128/mBio.00881-21) report the potential use of marine thraustochytrid metabolic thermodynamics in effective terpene engineering. Through comparative proteomics and metabolomics, as well as thermodynamic modeling, the authors demonstrated sodium-induced changes in thraustochytrid metabolism leading to a twofold increase in squalene accumulation. The differential abundances of the metabolic enzymes and metabolites, as well as higher respiration, indicated the metabolic shift from carbohydrate to lipid oxidation and increased ATP input to the mevalonate pathway and squalene synthesis. This breakthrough provides new important insights into microbial terpene metabolic engineering but above all displays thermodynamics as a valuable tool in metabolic engineering.

## COMMENTARY

Terpenoids represent a wide range of primary and secondary metabolites produced by plants, animals, and microbes. In some of these organisms, the mevalonate (MVA) pathway supplies isopentenyl diphosphate (IPP) and dimethylallyl diphosphate (DMAPP), which are further condensed to form a variety of terpene compounds, such as mono-, sesqui-, di-, tri-, and tertraterpenoids (1). Many of these molecules possess beneficial properties employed in various sectors of human activity, including the cosmetic and drug industries. For instance, the triterpenoid squalene is a well-known compound displaying a high potential for therapeutic applications, either used as an emulsion-like carrier for improved vaccine and drug delivery or due to its chemopreventive and immunostimulating effects (2). Currently, squalene is mainly extracted from shark liver and some vegetal sources (3), and its growing demand requires alternative supply strategies, such as microbe metabolic engineering. However, obtaining high production yields via this approach remains challenging due to limiting endogenous metabolic fluxes, competing pathways, and underperforming enzymes. In addition to the common optimization of metabolic fluxes and enzyme activity via gene expression fine-tuning, thermodynamics and understanding of carbon flux regulation between native primary and secondary metabolism were recently demonstrated as a valuable tool for efficient terpene engineering (4).

**FIG 1:**
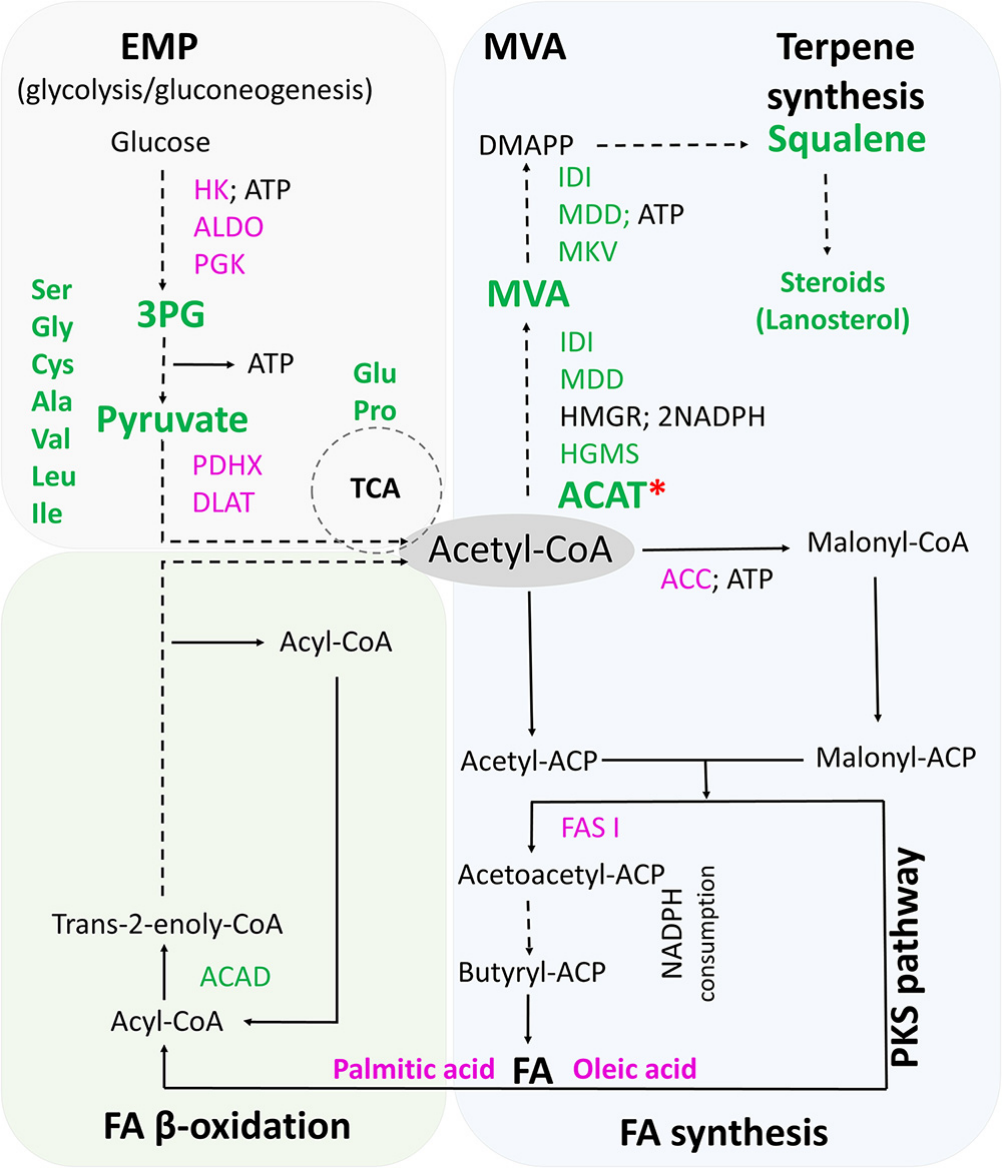
Sodium-induced changes in *Thraustochytrium* sp. metabolism (adapted from reference 4). The schematic pathways illustrate terpene synthesis from glucose or fatty acid (FA) β-oxidation, and the changes that occur in *Thraustochytrium* sp. metabolism under sodium (NaCl) supplementation growth conditions. Significantly increased levels of metabolites and enzymes are shown in green, while significantly decreased levels of metabolites or enzymes, determined by comparative proteomics and metabolomics, are shown in pink. For clarity, numerous steps were omitted (dashed arrows) to highlight the identified changes. The red asterisk indicates the rate-limiting enzyme in both growth conditions, determined by *in silico* analysis of local thermodynamics. EMP, Embden-Meyerhof-Parnas pathway; HK, hexokinase; ALDO, fructose-biphosphate aldolase; PGK, phosphoglycerate kinase; ACC, acetyl-CoA carboxylase; FAS I, fatty acid synthase I; ACAD, acyl-CoA dehydrogenase; MVA, mevalonate pathway; ACAT, acetyl-CoA acetyltransferase; HMGS, 3-hydroxy-3-methylglutaryl-CoA synthase; MVK, mevalonate kinase; MDD, mevalonate diphosphate decarboxylase; HMGR, 3-hydroxy-3-methylglutaryl-CoA reductase; IDI, 3-ketoacyl-CoA thiolase; 3PG, 3-phospho-d-glyceroyl phosphate; MVA, mevalonate; DMAPP, dimethylallyl diphosphate; PDHX, pyruvate dehydrogenase protein X component; DLAT, dihydrolipoyllysine-residue acetyltransferase component of pyruvate dehydrogenase complex; TCA, tricarboxylic acid cycle; PKS, polyketide synthase pathway.

In this recent article (4), the authors exploited the marine protist thraustochytrids (here *Aurantiochytrium* sp.), known to accumulate high levels of squalene and carotenoids in their natural sodium-rich environment (5, 6). In particular, this study reported a twofold increase in squalene accumulation, reaching 123.6 mg/liter under sodium (NaCl) supplementation growth conditions (4). To determine the mechanism behind the sodium-driven squalene accumulation, comparative proteomics were performed and highlighted differential abundances of metabolism-related enzymes between NaCl-supplemented and control growth conditions (Fig. 1). For instance, the main enzymes of the Embden-Meyerhof-Parnas (EMP) pathway (e.g., hexokinase [HK], fructose-biphosphate aldolase [ALDO], and phosphoglycerate kinase [PGK]) and the fatty acid (FA) synthesis pathway (acetyl coenzyme A [acetyl-CoA] carboxylase [ACC] and fatty acid synthase I [FAS I]) had significantly lower abundance under the high sodium growth condition, while the key enzyme of the β-oxidation pathway acyl-CoA dehydrogenase (ACAD) abundance increased 2.2-fold. These changes thus suggest a sodium-induced shift from carbohydrate to lipid oxidation, a lower fatty acid synthesis, and higher ATP/acetyl-CoA formation. On the other hand, the MVA pathway enzymes (e.g., acetyl-CoA acetyltransferase [ACAT], 3-hydroxy-3-methylglutaryl-CoA synthase [HMGS], mevalonate kinase [MVK], and mevalonate diphosphate decarboxylase [MDD]) showed a significantly increased abundance, demonstrating *per se* an enhanced metabolic flux toward terpene biosynthesis. Consistent with the proteomics analysis, the comparative metabolomic study displayed a 1.6- and 1.9-fold increase in mevalonate and squalene levels, respectively. In addition, despite a lower abundance of EMP pathway key enzymes, higher accumulations of glyceraldehyde 3-phosphate (3PG) and pyruvate (as well as of several downstream amino acids) were observed (Fig. 1) suggesting their synthesis via gluconeogenesis. Moreover, although the total fatty acid content did not significantly differ between the two growth conditions, several fatty acids, such as the most abundant palmitic acid and less abundant oleic acid decreased in the presence of supplemented sodium by 1.5- and 3.2-fold, respectively. These results support the rationale that in the presence of sodium, the cellular energy is preferentially generated from lipid degradation rather than glycolysis, which probably leads to a higher ATP/acetyl-CoA supply, thus favoring terpene biosynthesis. To strengthen their findings and identify the potential bottleneck(s) in squalene biosynthesis from glucose or short-chain fatty acid hexanoyl-CoA, the authors performed thermodynamic modeling and *in silico* analysis using the pathway analysis tool PathParser (7), which can predict the thermodynamically restrained metabolic step(s). Consequently, the thermodynamically highly unfavorable ACAT-mediated reaction, which requires acetyl-CoA as a substrate, was identified *in silico* as a rate-limiting step in both standard and high sodium growth conditions. In contrast, the measurement of acetyl-CoA levels at different time points between the two growth modes did not reveal any differences. Surprisingly, ATP levels were also found to be similar in the presence or absence of NaCl, suggesting an energy homeostasis-based regulation. Thus, cellular respiration, evaluated via the measurement of oxygen consumption, was significantly increased after 24 h in the sodium-supplemented cultures and pointed out ATP as a thermodynamic driving force for higher terpene production. In sum, the tremendous advances reported by Zhang and colleagues demonstrate that *Aurantiochytrium* sp. employs a sodium-induced mechanism to rewire its metabolism and adapt to the high sodium growth conditions. Such a process could be efficiently exploited as an additional tool to provide valuable and more specific insights for microbe metabolic engineering.

In this respect, the identified bottlenecks in heterologous production are routinely addressed via gene copy balance, the use of more active orthologs or engineered enzymes. In parallel, primary metabolism can be rewired to provide a higher cofactor supply and reduce the competition for common substrates (8). For instance, in yeast terpene biosynthesis, 3-hydroxy-3-methylglutaryl-CoA reductase (HMGR), which requires two NADPHs, is generally regarded as a limiting step instead of ACAT. However, for both enzymes as well as for HMGS, the expression of more active orthologs from Enterococcus faecalis was already shown to result in enhanced squalene titer (9). In addition to MVA pathway overexpression, yeast terpene engineering starts to make use of spatial reconfiguration of biosynthetic pathways, notably via enzyme peroxisome targeting, given the peroxisome intrinsic pool of fatty acid β-oxidation-derived acetyl-CoA (9, 10). For instance, after targeting the entire squalene biosynthetic pathway to Saccharomyces cerevisiae peroxisomes via the peroxisomal targeting signal PTS1, the squalene titer was improved 68-fold (650 mg/liter) (10), while another study demonstrated an improved biosynthesis of heterologous monoterpenoids via artificial peroxisomal localization of the MVA pathway and downstream heterologous enzymes in S. cerevisiae (9). Furthermore, the overexpression of several native genes involved in ATP import into the peroxisome, NADPH generation, and acetyl-CoA synthesis led to an additional 1.65-fold increase in squalene synthesis up to 1.3g/liter (10), which is coherent with the thraustochytrid thermodynamics. These strategies could additionally benefit from the thermodynamics-based data to reinforce rational pathway design. Indeed, implementing an identified thermodynamic driving force to direct the whole metabolism toward the desired compound production would undoubtedly lead to enhanced titers. Thus, in addition to the classical metabolic engineering strategies, the metabolic shift and local thermodynamic constraints identified in marine thraustochytrids have the potential to beneficially assist and refine rational terpene engineering. For instance, the enhancement of yeast FA β-oxidation and acetyl-CoA formation, which takes place in the peroxisome, could be assessed via the overexpression of corresponding enzymes to increase FA uptake from the FA-supplemented growth media (e.g., yeast protein kinase Ypk1p), activation (e.g., acyl-CoA synthases Fat1p and Faa1p/Faa2p/Faa3p/Faa4p) and degradation (e.g., acyl-CoA oxidase Fox1p) (11). In parallel, yeast respiratory metabolism could be favored to limit fermentation and increase ATP supply via altering glucose import (e.g., hexose transporter mutagenesis) (12). Globally, advances in thermodynamics data, in line with predictive engineering, systems metabolic engineering, and rational pathway design, presage a facilitated and more efficient native/heterologous pathway construction and optimization, and high-scale production.
